# Frequency-division multiplexer and demultiplexer for terahertz wireless links

**DOI:** 10.1038/s41467-017-00877-x

**Published:** 2017-09-28

**Authors:** Jianjun Ma, Nicholas J. Karl, Sara Bretin, Guillaume Ducournau, Daniel M. Mittleman

**Affiliations:** 1 School of Engineering, Brown University, 184 Hope Street, Providence, RI 02912 USA; 2 Institut d’Electronique de Microélectronique et de Nanotechnologie (IEMN), UMR CNRS 8520, Université de Lille 1, 59652 Villeneuve d’Ascq Cedex, France

## Abstract

The development of components for terahertz wireless communications networks has become an active and growing research field. However, in most cases these components have been studied using a continuous or broadband-pulsed terahertz source, not using a modulated data stream. This limitation may mask important aspects of the performance of the device in a realistic system configuration. We report the characterization of one such device, a frequency multiplexer, using modulated data at rates up to 10 gigabits per second. We also demonstrate simultaneous error-free transmission of two signals at different carrier frequencies, with an aggregate data rate of 50 gigabits per second. We observe that the far-field spatial variation of the bit error rate is different from that of the emitted power, due to a small nonuniformity in the angular detection sensitivity. This is likely to be a common feature of any terahertz communication system in which signals propagate as diffracting beams not omnidirectional broadcasts.

## Abstract (web summary)

There is growing interest in the development of components to facilitate wireless communications in the terahertz but the characterization of these systems involve an unmodulated input. Here the authors demonstrate multiplexing and demultiplexing of data streams in the terahertz range using a real data link.

## Introduction

The volume of wireless data traffic is increasing exponentially and will surpass 24 exabytes per month by ^1^2019. To accommodate this trend, future generations of wireless networks will require much higher capacity for data throughput. One favored solution is to operate at higher carrier frequencies, beyond 100 GHz^2–5^. Recent years have witnessed rapidly growing interest in the development of components to enable wireless communications in the terahertz (THz) range. One of the earliest examples is modulators, first discussed almost 20 years ago^6^, with rapid improvements continuing to be reported^7–10^. Other examples include power splitters^11, 12^, filters^13, 14^, phase shifters^15^, beam-steering devices^16–18^, passive reflectors for engineered multipath environments^19, 20^, and multiplexers and demultiplexers (mux/demux)^21, 22^. Despite these efforts, many important components of such networks remain at a very immature stage of development, including components for mux and demux. Mux and demux of non-interfering data streams is universally employed in existing communication systems and, in combination with advanced modulation schemes^23^, can be an efficient method to achieve the eventual data rate target of Tb/s. In the THz range, where frequency bands may not be continuous over a broad spectral range due to atmospheric attenuation^24^ or regulatory restrictions^25^, frequency-division multiplexing is even more of a compelling need.

We have recently proposed an architecture for waveguide-to-free space mux/demux based on a leaky waveguide^21^. This concept exploits the highly directional nature of THz signals, which are much more like beams than omnidirectional broadcasts. A particular client in a network would be assigned a spectral band based on its location, such that only signals within that spectral band are sent to the location of the particular client. The device can accommodate mobility by tuning the carrier frequency to account for changes in the client location; this process would likely rely on beam-sounding techniques using legacy bands at lower frequencies^26^. Alternatively, multiple clients can be served simultaneously by mux/demux of multiple signals lying in distinct frequency bands.

The operating principle of the leaky-wave device is straightforward. It is based on a metal parallel-plate waveguide (PPWG), which has proven to be a versatile platform for manipulation of THz signals^27, 28^. The waveguide has a narrow slot opened in one of the metal plates, which (in the demux configuration) allows some of the guided wave to leak out into free space. Similar leaky-wave designs have been used in the RF community for many years^29^, but their use in the THz range has so far been limited^21, 30, 31^. The frequency of the emitted radiation at a given angle is determined by a phase-matching constraint:

1 $${k_0}\cos \phi = {k_{{\rm{PPWG}}}},$$

where *k*_0 = _2π*v*/*c*_0_ is the wave vector for free space with *v* as the frequency of the signal and *c*_0_ as the speed of light in vacuum. *ϕ* is the propagation angle of the free-space mode relative to the waveguide propagation axis. The frequency-dependent propagation constant for the lowest-order transverse-electric (TE_1_) mode of a PPWG is^27^:

2 $${k_{{\rm{PPWG}}}} = {k_0}\sqrt {1 - {{\left( {\frac{{{c_0}}}{{2bv }}} \right)}^2}} ,$$

where *b* represents the plate separation. Substituting Eq. (2) into Eq. (1), the phase-matching condition results in an angle-dependent emission frequency:

3 $$v = \frac{{{c_0}}}{{2b\sin \phi }}.$$

For an incoming wave, the situation is simply reversed; an incident wave at a given frequency only couples into the waveguide if it arrives at the appropriate angle determined by Eq. (3). Thus, the design supports both mux and demux capabilities.

Although this initial study of a mux/demux device, and the other device demonstrations mentioned above, all represent significant advances in THz signal processing, it is important to note that these measurements have usually been performed in isolation with an unmodulated continuous-wave or pulsed time-domain source. Characterization of the performance of these devices in the context of a communication system, using data modulated at high bit rate, has for the most part not been demonstrated, and little consideration has yet been given to the enormous challenge of integration into a larger system. Meanwhile, there have also been several recent single-input single-output (SISO) THz link demonstrations^3, 23, 32–35^, which have achieved impressive data rates but have so far not progressed to the integration of any of the aforementioned signal processing components.

In this article, we report an attempt to bridge this conceptual gap, with the characterization of a THz mux/demux subsystem^21^ in a real THz data wireless link. We use modulated data to characterize bit error rates and power penalties for this subsystem, as a function of data rate and source power. We achieve single-channel error-free mux/demux at rates up to 10 gigabits per second (Gb/s), as well as the first report of mux/demux of two independent real-time video broadcasts, and the demux of two frequency channels with an aggregate data rate of 50 Gb/s. This work represents the first simultaneous mux/demux of real data flows in the THz range.

## Results

### Characterization of bit error rate

**Fig. 1:**
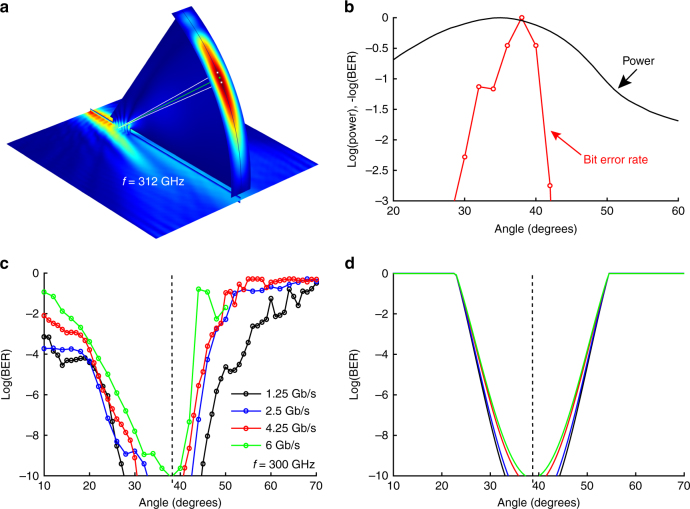
Demultiplexing of modulated THz channels for different data rates. **a** A 3D numerical simulation (finite element method), of a single-frequency input wave (*f* = 312 GHz) propagating in the waveguide (*b* = 0.733 mm) and then radiating into the far field through a slot in the top plate. The *horizontal plane* shows the intensity in a plane centered between the metal plates (i.e., inside the waveguide). The *vertical* (out of plane) *arc* shows the radiated power as a function of angle. The *solid green line* indicates the angle predicted by Eq. (3) for the parameters used in this simulation. The two *solid white lines* on either side of the *green line* show the predicted angles for frequencies of 302 GHz and 322 GHz, corresponding to the ±1st-order sidebands for a modulation data rate of 10 Gb/s. The angular spread of these sidebands is smaller than the angular width of the carrier wave diffracting through the slot. **b** Measured angular distributions for the power (*black curve*) and bit error rate (*BER*, *red symbols*), for an input frequency of 300 GHz and a modulation rate of 6 Gb/s. Both are normalized to unity and plotted on a log scale (BER plotted as the negative log), to facilitate comparison of the angular widths. **c** Measured real-time BER performance of the THz link coupled out from the slot, as a function of the angular position of the detector, for a 300 GHz carrier wave. Here, the plate separation *b* is 0.8 mm and slot width is 0.7 mm. Results for several different data rates all show the same optimum angle of 38.7° independent of the data rates (indicated by the *vertical dashed line*), though the angular width varies slightly with data rate. **d** A model calculation of the effect of a non-uniform angular detection sensitivity on the BER, which qualitatively reproduces the observed results. These *curves* assume a specific (parabolic) form for the angular detection filter, but otherwise contain no free parameters (see Supplementary Note 1 for details). In this plot, the colors correspond to the same data rates as in (**c**)

The numerical simulation in Fig. 1a illustrates the performance of the leaky waveguide in a demux configuration, for a single-frequency (unmodulated) input wave, first propagating inside the waveguide and then radiating into free space and producing a diffracting beam in the far field at an angle determined by Eq. (3). The *solid green* and *white lines* added to this simulation show that the angular spread of first-order modulation sidebands is expected to be smaller than the size of the diffracting carrier wave, even up to 10 Gb/s. This suggests that a detector with sufficient aperture to collect most of the carrier wave will also capture the modulation information required for signal transmission. However, our experimental results, described below, reveal a surprising sensitivity of the signal quality to the angular position of the receiver, resulting from a small angular nonuniformity in the detection sensitivity.

We first explore the performance of the device in the demux configuration, with a single data-modulated input wave. We generate the THz signal by photomixing two infrared optical signals modulated using an optical modulator, resulting in a an amplitude-modulated signal (amplitude shift keying, ASK) with a carrier frequency determined by the optical frequency difference. This signal is coupled into the waveguide with an input power of about −10 dBm. The waveguide consists of two flat steel plates, with a plate separation of *b* = 0.8 mm and a length of 40 mm. The input aperture of the waveguide is tapered to improve the input coupling efficiency^36^. The slot in the top waveguide plate has a length of 28 mm and a width of 0.7 mm, and begins 5 mm beyond the input face of the waveguide. The signal radiated from the slot is collected by a Teflon lens (*f* = 25 mm) and focused onto a Schottky diode receiver. The collection and detection system is mounted on a rotation arm, to characterize the output as a function of the angular position of the receiver. After electrical amplification, the bit error rate (BER) is determined in real-time, i.e., without any off-line processing.

Figure 1 shows typical results for an input wave of 300 GHz (which, for the given value of *b*, corresponds to an output angle of 38.7°). Figure 1b shows a comparison of the angular distribution of the power to the angular dependence of the BER measured under identical conditions. Figure 1c displays the BER at different receiver angles, for several different data-modulation rates, all with the same carrier frequency.

This figure demonstrates several important results. First, we observe error-free data transmission through the demux device (BER < 10^−10^) for all data rates, proving that the propagation through the waveguide does not introduce excessive signal loss or distortion due to dispersion. This is consistent with previous work demonstrating the low-loss and low-dispersion characteristics of TE_1_ mode propagation in parallel-plate waveguides^27, 37^. We also note that the optimum BER and maximum power are always obtained at the same angle, regardless of the modulation rate. This is not surprising, as the angle is determined by the carrier frequency and the plate separation, according to Eq. (3).

The most surprising aspect of Fig. 1b and c involves the angular widths of the BER curves, which are all in the vicinity of just 2 or 3° (FWHM). This is considerably smaller than the measured angular width of the power distribution (as shown clearly in Fig. 1b), and also smaller than angular aperture of our collection optics. Moreover, at a given BER, the widths of the *curves* in Fig. 1c vary slightly with data rate, becoming somewhat narrower as the data rate increases. This strong and anomalous angular dependence suggests that the BER is significantly influenced by the angular sensitivity of the detection of modulation sidebands, which co-propagate with the carrier frequency (at slightly different angles, as shown in Fig. 1a), in a diffraction-limited beam.

Using a simple model for the angular filtering of the receiver, we can qualitatively understand both the observed angular widths and the data-rate dependence shown in Fig. 1c. We imagine that, regardless of the details of the detection system, its sensitivity (when it is located at a particular angular location) is a slowly varying function of the propagation angle of the THz signal, with a maximum sensitivity when the beam propagation angle is equal to the detector angle so that the beam hits the center of the detector. If the detector is moved so that it is not centered on the diffracting beam (i.e., at the angle determined by Eq. (3) for the carrier frequency), then positive-modulation sidebands and negative-modulation sidebands will not be detected with equal sensitivity. Even if this spectral asymmetry is small, it will lead to a decrease in the overall signal-to-noise of the detection, and thus a degrading of the BER. We note that this effect will not impact the detection of the overall signal power, which explains why the angular width of the power curve is significantly larger than that of the BER curve in Fig. 1b. Modulation at a higher data rate produces sidebands that are more widely spaced in frequency and therefore also in angle. These are more sensitive to the angular filtering as they sample the filter at larger angles away from the optimal central angle. Thus, the angular degradation of the BER is more rapid at higher modulation rates, consistent with our observations. Figure 1d shows the results of a simple model calculation, using an assumed parabolic form for the angular-filter function, which qualitatively reproduce the observed angular widths and also the trend with data rate (see Supplementary Note 1 for details). We note that the BER values estimated from this model change substantially within a small angular range, even though the assumed spectral filter is quite flat, varying by only about 1% within ± 10 GHz of the central frequency.

Given the highly directional nature of THz signals, this angular sensitivity is likely to be a quite general feature of any THz wireless network in which frequency multiplexing is used and in which beam widths are diffraction-limited. This result, which would not have been observed using an unmodulated THz source, has important implication for the trade-off between receiver aperture and data rate, and also for the design of antenna configurations in optimal multiple in/multiple out (MIMO) architectures^3, 38^.

**Fig. 2:**
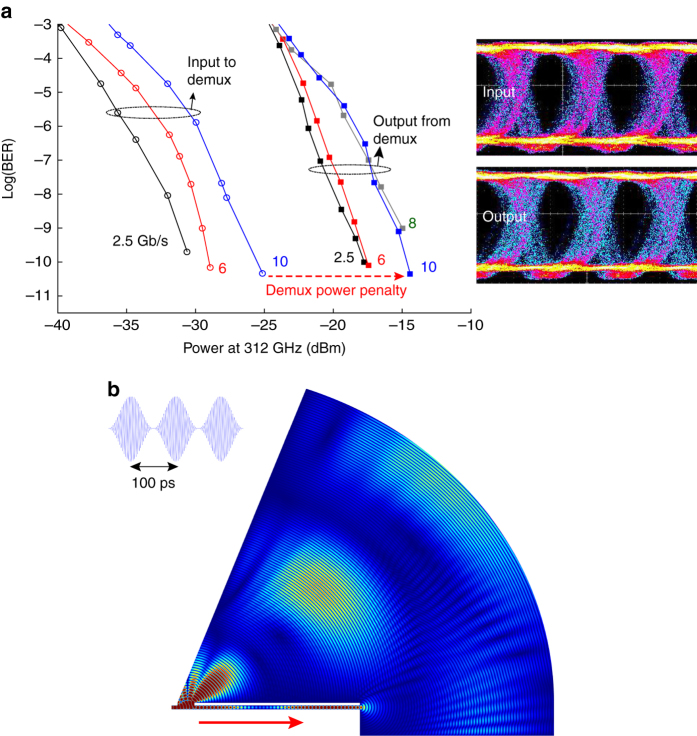
Demultiplexing of modulated THz channels as a function of detected power. **a** Measured real-time BER performance of the THz link as a function of the THz power at the receiver under different data rates up to 10 Gb/s. Values are recorded both before the demultiplexer (*left set* of *curves*), and also after demultiplexing (*right set* of *curves*) with the detector fixed at the optimum angular position for the carrier frequency of 312 GHz. Data rates are shown next to each *curve*, in Gb/s. Typical eye diagrams are shown for the input and demultiplexed links at a data rate of 10 Gb/s, both showing error-free transmission (*BER* < 10^−10^). Before demultiplexing, all the *curves* have about the same slope. But after the device, the slope changes for the higher data rates (8 and 10 Gb/s), due to scattering of residual radiation at the output end of the waveguide. **b** One frame from a two-dimensional numerical time-domain simulation movie, depicting the scattering phenomenon, which leads to inter-symbol interference at higher data rates, as discussed in the text. The *inset* (*upper left*) shows the input waveform for the simulation, which is a 300 GHz carrier wave modulated so that a pulse of radiation enters the waveguide every 100 ps. The waveguide is at the *bottom left*, where the *red arrow* indicates the propagation direction for the guided wave. Interference fringes are clearly evident due to interference between the bit emerging from the far end of the waveguide and the previous bit, which radiated through the slot

Another important parameter is the insertion loss, which induces a power penalty for error-free operation. To explore this issue, we compare the measured BER values for demuxed signals (at the optimal receiver angular location) to those measured without demux; in that latter case the detector is placed directly at the location of the demux input port, bypassing the demux waveguide entirely. This result, shown in Fig. 2a, quantifies the power penalty induced by the demux. For example, at 10 Gb/s, the penalty is about 10 dB. These measurements were obtained for a carrier frequency of 312 GHz, and various data rates, up to 10 Gb/s (10 G Ethernet data rate) as indicated in the figure. *Insets* show the eye diagrams for a modulation rate of 10 Gb/s, both before and after demultiplexing. The eye opening becomes a little bit narrower after demultiplexing due to the power penalty, but it is still possible to obtain error-free transmission at all data rates, reaching a BER below 10^−10^. This penalty is probably due almost entirely to the efficiency of the coupling into and out of the waveguide, and not to propagation losses or dispersion inside the waveguide, which are known to be small^37^.

We also observe that the slope of the demuxed BER curves changes for higher data rates (above 6 Gb/s), indicating an increased noise level at these higher modulation rates. We speculate that this increased noise arises from signals emerging from the far end of the waveguide (rather than from the slot, as intended). The impedance mismatch to free space is not large^39^, so most of the remaining power is emitted into air, and then can scatter from this abrupt waveguide termination to cause interference at the detector. Such scattered signals are delayed by their extra travel time inside the waveguide. If this delay exceeds the duration of a single bit, then this coherent interference can leak over into the subsequent bit, thus degrading the eye diagram. Therefore, one could expect a higher BER for signals with data-modulation rate larger than a certain threshold value determined by the inverse of the extra travel time of the scattered interference signal. The phase delay inside the waveguide, roughly 190 ps, indicates a threshold value near 5 Gb/s for this inter-symbol interference (ISI) effect, which is close to what is observed experimentally in Fig. 2a. This idea is supported by the numerical time-domain simulation shown in Fig. 2b, for a bit period of 100 ps, (corresponding to a data rate of 10 Gb/s). This simulation is somewhat limited in accuracy as it is only a 2D simulation; nevertheless one can clearly see the fringes due to ISI between a bit emerging from the slot and one emerging from the end of the waveguide.

### System demonstrations

**Fig. 3:**
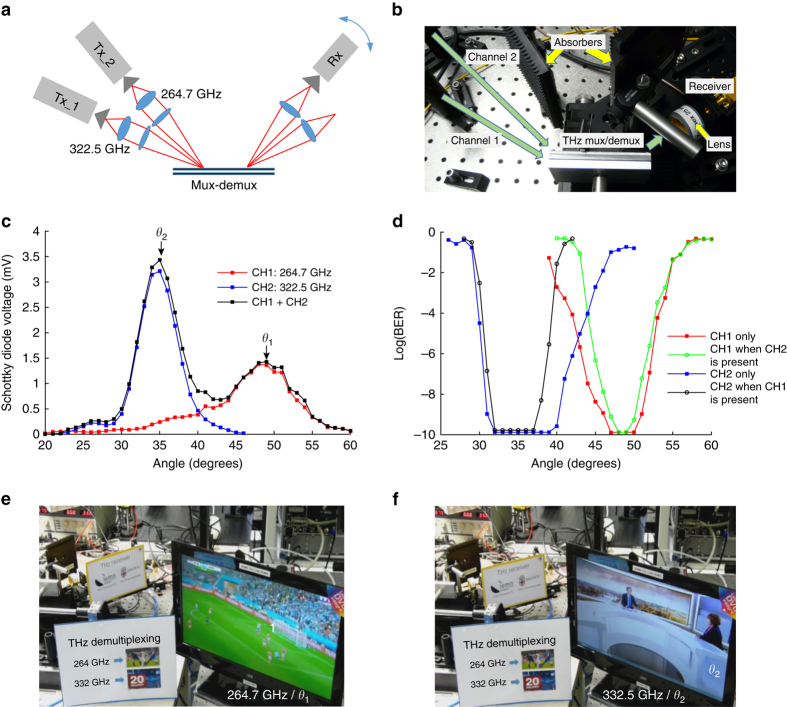
Schematic diagram and multiplexing/demultiplexing of two THz channels. **a** Schematic showing the measurement setup, with two different transmitters at 264.7 GHz and 332.5 GHz at fixed angular positions, and with the receiver mounted on a pivoting rail to vary the measurement angle. Power pattern and BER performance for both real-time links at 264.7 GHz and 332.5 GHz are measured after mux-demux with data rate at 1.5 Gb/s. **b** View of the mux-demux in the experimental setup. **c** Power pattern measured when channel 1 (264.7 GHz) is on while channel 2 (322.5 GHz) is off (*red curve*), channel 2 is on, whereas channel 1 is off (*blue curve*), and both channels are on (*black curve*). **d** BER performance for channel 1 only (*red*), channel 2 only (*blue*), channel 1 when channel 2 is on (*light green*) and channel 2 when channel 1 is on (*dark green*). Error-free operation can be achieved in both channels even with both signals on. (**e**, **f**) Two real-time videos (HD-TV broadcast) transmitted by the two THz links at 264 GHz and 332.5 GHz, each with a data rate of 1.5 Gb/s. The video signals are taken from two different TV broadcast channels and connected to the transmitters. In the monitor connected to the detector, the channel switches when the angular position of the receiver changes. This THz mux/demux can be observed in operation in the Supplementary Movie, showing excellent stability and reproducibility

To demonstrate the real-time mux and demux operation, we use two independent transmitters as shown in the schematic in Fig. 3. In this case, one channel is the photomixer-based THz source described above, and the other one is a frequency multiplication chain. These two signals with carrier frequencies of 264.7 GHz (channel 1, electronic source) and 322.5 GHz (channel 2, photomixer), are both amplitude-modulated (ASK modulation, as above) with independent bit patterns, both at a data rate of 1.5 Gb/s. The input powers were adjusted to reach a similar performance on the two signals and correspond to around −10 dBm in each channel incident on the mux input. In this case, the waveguide consists of a longer pair of plates (length = 80 mm) with two slots in the top plate, on opposite ends. We use one of the slots to couple two different signals into the waveguide (mux), and the other slot to couple them out (demux). In this measurement, the effective propagation distance for the two signals inside the waveguide is 14 mm. The input angles of the two signals into the first slot are adjusted according to the criterion of Eq. (3), to optimize the efficiency of input coupling into the waveguide. At the output, the receiver is rotated through a range of angles to characterize the angular distribution of the output, as in Fig. 1. We measure both the power (Fig. 3c) and the BER (Fig. 3d) as a function of angle, for each transmitter individually and also when both signals are in the waveguide at the same time. Figure 3c shows that the optimal output angles are again consistent with the prediction of Eq. (3). Figure 3d shows that the BER is <10^−10^ for both channels, whether or not the other channel is present. In other words, we achieve error-free mux and demux for each channel, whether or not the other channel is simultaneously propagating in the waveguide. The small changes in each BER curve when the other channel is present can be understood by noting the small overlap between the two demuxed beams as show in Fig. 3c. Nevertheless, it is clear that error-free mux-demux can be achieved for both channels. We further demonstrate this remarkable result by modulating the two channels using real video data from two different television broadcasts. When the receiver is rotated from one optimum angular position to another, the received video shown on the monitor switches from one channel (Fig. 3e) to another (Fig. 3f).

**Fig. 4:**
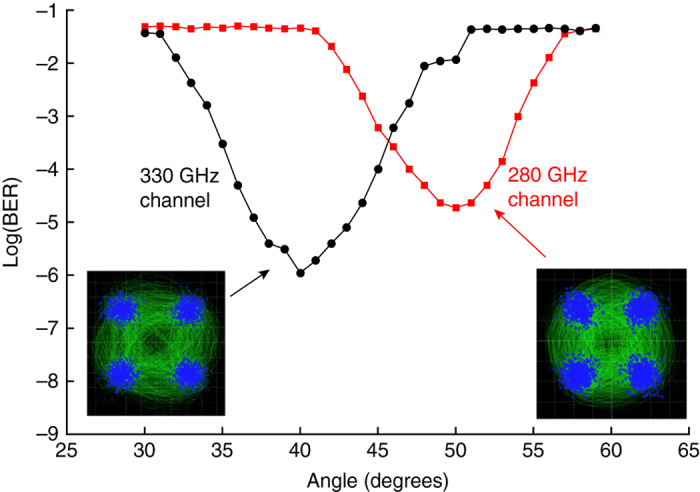
Demux of two QPSK-modulated channels. BER vs. angle for two channels at 280 GHz and 330 GHz, both modulated at 12.5 Gbaud (corresponding to 25 Gb/s in each channel), for an aggregate throughput of 50 Gb/s. To preserve the QPSK phase information, signals were detected using a Schottky-based sub-harmonic mixer with the output analyzed on a real-time high-bandwidth oscilloscope. In both cases, the optimum BER is well below the threshold for forward error correction. The *insets* show the constellation diagrams measured for each channel. The *vertical dashed lines* show the predicted positions of the BER minima for the two channels, according to Eq. (3)

Finally, we explore the efficacy of higher order modulation schemes, which can provide increased data rates while using less spectral bandwidth. For this measurement, the photomixer THz source is driven by an optical signal modulated using quadrature phase shift keying (QPSK) at 12.5 Gbaud. In this case, two QPSK-modulated carrier signals, each carrying 25 Gb/s of data, are generated in the photomixer at frequencies of 280 and 330 GHz. These are simultaneously injected into a waveguide in a demux configuration (plate separation = 0.7 mm, slot width = 0.8 mm), and the two outputs were measured independently as a function of angle. To preserve the phase information contained in the QPSK signal, we detect the signals using a sub-harmonic mixer. The down-converted signals are analyzed to recover the constellation diagrams and BER performance for both channels. This result, shown in Fig. 4, demonstrates demux of two signals with an aggregate data rate of 50 Gb/s, with acceptable BER of ~10^−5^ or better for both channels. Although not error-free, the BER is still well below the threshold for forward error correction (typically 2 × 10^−3^). The degraded BER relative to the results shown in Fig. 3 are probably due to the same effect of interference with scattered light mentioned above, which would be expected to have an increasing impact with increasing data rate.

## Discussion

In summary, we have explored the performance of a leaky-wave device for multiplexing and demultiplexing in THz wireless links, using a realistic system configuration with the modulated data. We obtain error-free data transmission through the demux device for all data rates up to 10 Gb/s, which demonstrates that neither insertion loss nor waveguide dispersion are limiting factors in the operation of this mux/demux configuration. We characterize the power penalty when the wave propagates through the waveguide. This effective insertion loss results mainly due to the coupling efficiency between free space and the waveguide mode, and can therefore be further optimized by tailoring the waveguide input and/or the slot width.

Because of the strongly directional and diffraction-limited nature of THz signals, the measured bit error rate depends on the angular location of the detector, which changes with the data-modulation rate. This new phenomenon can be understood by applying a relatively simple filtering model. As any network operating above 100 GHz is almost certain to exhibit narrow diffraction-limited beams, this may be the limiting factor in achievable data rate, for a given single-point receiver aperture. On the other hand, in a MIMO configuration different antennas in an array may receive different subsets of the total spectral information in a signal. This presents an interesting challenge in the optimal detection and demodulation of demuxed signals, which could overcome the limitation imposed by a diffracting beam with spectral sidebands.

In addition, we demonstrate the effectiveness of this mux/demux approach by operating two independent wireless links at 264.7 GHz and 332.5 GHz to demonstrate real-time mux and demux, for simultaneous error-free transmission of two video signals with ASK modulation, as well as the demux of two QPSK-modulated signals with aggregate data rate of 50 Gb/s. Our results clearly suggest that two frequency channels is not the limit; additional channels could be added for increased aggregate throughput. In our earlier work^21^, we modeled a six-channel configuration with equal 20 GHz-wide channels spaced over 150 GHz of spectrum. This model configuration seems to be feasible, although an experimental realization would require an array of sources that are probably not yet available in any one laboratory. The practical limit on channel number will likely be determined by the size and positioning of coupling optics. We note that this mux/demux configuration can also accommodate mobility, with continuous tuning of the carrier frequency as a user moves and the angle between the waveguide axis and the user changes. This would obviously require a continuously tunable or very broadband THz source, which may be feasible using SiGe BiCMOS process technology^40^.

It is interesting to note the contrast with free-space optical (FSO) networking, another feasible approach to achieving wireless links with Tb/s throughput. FSO links can also employ frequency multiplexing, and like a THz link, the signals propagate as directional beams, not omnidirectional broadcasts^41^. However, the wavelength-dependent diffraction effects described here would not be expected to manifest themselves in FSO systems. The relevant parameter here, to determine the significance of diffraction effects, is the spacing between adjacent frequency channels d*v*, as a fraction of the average carrier frequency *v*_0_. In a typical frequency-multiplexed FSO system^41^, this fractional spacing d*v*/*v*_0_ is quite small, on the order of 10^−4^. In contrast, for our system demonstration (Fig. 4), this parameter is almost three orders of magnitude larger. Thus, diffractive spreading of the carrier wave (and all modulation sidebands) is a significant phenomenon in THz systems, and is irrelevant in FSO links where all of the multiplexed signals co-propagate with parallel wave vectors. Beam diffraction can be both a challenge and an advantage; for example, beam misalignment due to, e.g., atmospheric turbulence is a huge challenge for long-distance FSO links with tightly collimated beams, but has essentially no impact on THz links^42^. Of course, THz links also afford the substantial advantage that coherent phase-sensitive detection is relatively straightforward, which enables MIMO architectures that would be exceedingly challenging to implement using visible or near-infrared light sources.

Finally, by noting the differences between simple power measurements and BER data, we emphasize the fact that the study of THz signal processing devices using modulated data in realistic configurations can reveal new information about their characteristics. In many cases including this one, this information cannot be readily obtained using conventional measurements with an unmodulated continuous-wave or pulsed time-domain source. Thus, measurements using data-modulated signals will be crucial for optimizing device performance in communication networks.

## Methods

### Measurement setup

The THz link performance measurement setup consists of two THz sources, one based on photomixing technologies (332.5 GHz) and the other on a frequency multiplexer chain (264.7 GHz) with a tunable output in the 260–330 GHz frequency band. Detection is achieved using a zero-biased Schottky diode broadband intensity detector associated to RF amplifiers (amplification bandwidth of 12 GHz, which determines the overall system bandwidth) to drive the BER tester (N4903A J-BERT from Agilent Technologies, with the option A01/C13). The average output power of the two THz sources is tunable and adjusted to reach the best driving signal for the Schottky diode and RF detection for BER measurements. We verified that the two beams contain almost same power, by comparing the rectified voltages at Schottky output at the two optimal angles. For the THz signal intensity detection investigated in this study, we keep the THz power low enough to avoid saturating the detector, to optimize the signal-to-noise ratio of the detected signals. Last, we use absorbers to prevent detection of spurious signals that could leak out of the far end of the waveguide and scatter towards the receiver, or that could couple from the source directly to the receiver without propagating through the waveguide. We found that these absorbers were necessary in order to measure error-free performance, due to the effects of scattered radiation. Indeed, our efforts to block scattered signal at the waveguide output may require further improvement, as suggested by the data of Fig. 2. This emphasizes the extreme sensitivity of the BER to interference from scattered signals, which must be addressed with some care.

For the experiments employing QPSK modulation, an optical signal is modulated using a dual-nested Mach-Zender modulator before the photomixing process to generate the dual THz signal at 280 and 330 GHz. Two arbitrary waveform generators are used to create two baseband non-return-to-zero (NRZ) data signals for the in-phase and quadrature date flows. For detection, the dual frequency THz signal is down-converted in a Schottky-based sub-harmonic mixer to below 40 GHz. The output is amplified and then detected by a wide bandwidth oscilloscope (Tektronix DPO70000SX ATI, bandwidth of 70 GHz). The two QPSK signals corresponding to the two down-converted THz channels are analyzed to recover the two 25 Gb/s modulated data and the corresponding constellation diagrams.

### Simulations

Finite element method (FEM) simulation results were performed using COMSOL Multiphysics 5.2 with the RF module. Figure 1a shows a typical simulation result. For this figure, a perfect electric conductor (PEC) was used for the waveguide boundaries, with perfectly matched layers (PML) to absorb at the waveguide output. Scattering boundaries were used on the waveguide edges and on the upper air boundaries. A port boundary was used for the waveguide incidence, exciting the TE_1_ mode with a spot size of 1 mm. The waveguide width and length were 25 mm, with a 0.733 mm plate separation. The waveguide slot is 0.7 mm in width and 3 mm long and is located 4 mm from the front of the waveguide. The air above the waveguide is a 60° circle section extrusion with a radius of 22 mm and a width of 3 mm. Tetrahedral elements were used to mesh the geometry with a total of 4,831,496 domain elements. This simulation was solved at 312 GHz using the GMRES iterative solver.

The result shown in Fig. 2b was obtained using COMSOL Multiphysics 5.2 with the RF module in a transient finite difference time-domain simulation. PEC was used for the waveguide boundaries, with scattering boundary conditions to absorb in free space. A scattering boundary is used for the waveguide input. For exciting the parallel-plate waveguide TE_1_ mode, an amplitude-modulated signal was used as the input with a carrier frequency of 300 GHz, and with a modulation corresponding to a 10 Gbps data rate. The waveguide length was 33 mm, with a 0.8 mm plate separation. The waveguide slot is 3 mm long and is located 1 mm from the front of the waveguide. The air above the waveguide is a 60° circle section with a radius of 66 mm and a sector angle of 70°. Tetrahedral elements were used to mesh the geometry with a total of 465,048 domain elements. The simulation was solved to 300 ps with 0.1 ps time resolution.

### Data availability

All relevant data are available from the authors.

## Electronic supplementary material

Supplementary Information

Description of Additional Supplementary Files

Supplementary Movie 1
