# Supplementary material for: Frequency-division multiplexer and demultiplexer for terahertz wireless links
Source: Nat Commun. 2017 Sep 28;8:729. doi: 10.1038/s41467-017-00877-x (PMC5620079; doi:10.1038/s41467-017-00877-x)
Supplement: Supplementary file 1 — Supplementary Information [file 41467_2017_877_MOESM1_ESM.pdf]

Supplementary Note 1: Details of the model calculation shown in Figure 1(b)

We begin with a continuous wave carrier, at frequency f_0 = 300 GHz. We then modulate this carrier using ASK modulation, producing sidebands symmetrically about f_0. These sidebands are spaced by the frequency of the modulation, Δf (in our experiments, this has various values between 1.25 GHz and 6 GHz). Thus, we generate sidebands centered at $\pm\Delta f$, $\pm 2\Delta f$, $\pm 3\Delta f$, etc.

We then detect this signal using an incoherent detector (power only, not phase). This detector has a frequency-dependent detection sensitivity, due to the fact that different frequencies emerge from the demux at different angles. Thus, the detector's spectral sensitivity is determined by its angular aperture relative to the output of the leaky-wave antenna, and the angular acceptance of the horn antenna into which the radiation is coupled. For this analysis, we model this angular sensitivity using a simple model function which captures the essential behavior, and we neglect any other frequency dependence in the detection process.

This frequency-dependent detection is essentially a filter function, which peaks at a frequency determined by the angular position of the receiver, and falls off on either side. We assume that the filter is symmetric in angle, and we assume a parabolic shape as a function of angle ϕ, so that:

$$G(\phi) = 1 - \beta(\phi - \phi_C)^2 \quad (1)$$

where ϕ_C is the angle at which the center of the receiver is located and where β is a scale parameter which determines the angular width of the detection. We choose the value of β such that the width of the function is equivalent to the angular acceptance aperture of the detector in our experiments. A value of β = 14.3 (for ϕ and ϕ_C expressed in radians) gives an angular width of $2\sqrt{1/\beta} = 0.53$ radians.

This filter function $G(\phi)$ is expressed in a linear (not dB) scale, so that the fraction of power at frequency f emitted by the demux and detected by the receiver is simply given by $G(\phi(f))$. Of course, there is a one-to-one (but not linear) mapping between angle and frequency, as expressed by Eq. (3) in the text. At the center of the filter, $G(\phi_C) = 1$ (i.e., the signal is not degraded at all by angular effects). For angles ϕ far from ϕ_C, this analytic form for $G(\phi)$ becomes negative. For these values of ϕ, we assume that there is zero power detected (i.e., the signal simply misses the detector), and so we set $G = 0$.

We note that this is a rather flat filter, in the sense that $G(\phi)$ does not change very much in the range of angles that define the modulation bandwidth. For example, if the filter is centered at the angle corresponding to the carrier frequency f_0, then sidebands located ±10 GHz away from the carrier frequency experience less than a 1% decrease in detection sensitivity (i.e., $G_{\pm 10\text{ GHz sideband}} \approx 0.99$).

For a given detector angle ϕ_C, we can compute the value of G for the two lowest-order sidebands $f_0 \pm \Delta f$, located at angles ϕ_+ and ϕ_-, respectively:

$$G(\phi_\pm) = 1 - \beta\left(\sin^{-1}\left(\frac{c_0}{2b(f_0 \pm \Delta f)}\right) - \phi_C\right)^2 \qquad (2)$$

Since $G(\phi_+) \neq G(\phi_-)$, the two sidebands are detected asymmetrically. This asymmetric situation can be described as a superposition of amplitude modulation (for which the two sidebands are exactly in phase) and phase modulation (for which the two sidebands are π out of phase). This superposition is governed by a pair of linear equations, which describe the fact that:

(a) for the larger of the two sidebands (say, $G(\phi_+)$), the amplitude and phase modulated signals coherently add; and

(b) for the smaller of the two (say, $G(\phi_-)$), the amplitude and phase modulated signals coherently subtract.

Thus, we have:

$$\begin{aligned} G_+ &= A + P \\ G_- &= A - P \end{aligned} \qquad (3)$$

and therefore the amplitude-modulated portion of the signal has a relative amplitude of

$$A = \frac{G_+ + G_-}{2} \qquad (4)$$

Our detector, which is insensitive to phase, detects only this amplitude-modulated fraction of the signal. Thus, an asymmetric detection leads to a degraded signal-to-noise. Assuming that the noise is independent of angle, we determine the change in the signal-to-noise induced by this angular filtering as the square of the AM-modulated fraction of the signal:

$$\Delta_{S/N} = A^2 \qquad (5)$$

We note that, due to the nonlinear mapping between angle and frequency, this change in the signal-to-noise does not vanish even if the detector is centered precisely at the angle corresponding to f_0 (i.e., since the filter is symmetric in angle, it is therefore not perfectly symmetric in frequency). However, for a reasonable filter width, this effect is quite small; even for a modulation rate of Δf = 10 Gb/sec, it amounts to only about a 1% decrease in the value of A at f_0. Thus we may ignore this effect in our analysis.

We must now convert this signal-to-noise degradation into a degradation in the BER. For incoherently detected ASK modulation, the BER is related to the energy/noise ratio by [1]:

$$
\begin{aligned}
BER &\propto e^{-E/4N} \cdot erfc\left(\sqrt{\frac{E}{2N}}\right) \\
&= e^{-A^2/4} \cdot erfc\left(\frac{A}{\sqrt{2}}\right)
\end{aligned}
\qquad (6)
$$

This represents the change in BER as a function of detector angle. However, this analysis ignores the fact that the BER depends on input power, even if the detector is positioned at the optimal angle. Since the BER depends on power level, it is necessary to normalize the minimum value of this function to the appropriate BER at the power level used in the measurements. This power dependence can be extracted from the data shown in Fig. 2. We note that, at a given input power, the log(BER) depends on the data rate (i.e., on Δf), in a fashion which is approximately linear with data rate. Thus, we normalize the computed BER values according to:

$$
\left[\log\left(BER\right)\right]_{norm} = \log\left(BER\right) \cdot \frac{N\left(\Delta f\right)}{\log\left(BER\right)_{\min}} \qquad (7)
$$

where $N(\Delta f)$ is a normalization factor that varies linearly with Δf. From Fig. 2, we extract values for this linear normalization factor: $N\left(\Delta f\right) = 0.74\Delta f - 15.2$. Also, $\log(BER)_{\min}$ is the minimum value of the computed BER curve in the absence of normalization, given by:

$$
\log\left(BER\right)_{\min} = \log\left[e^{-1/4} \cdot erfc\left(\sqrt{\frac{1}{2}}\right)\right] \approx -0.607 \qquad (8)
$$

This data-rate-dependent normalization procedure is reasonable because in this analysis we are only interested in the change in BER due to a change in the angular position of the receiver, not in the absolute BER value at any given angle.

The result of this model calculation, a plot of $\log(BER)_{norm}$ versus ϕ_C for different values of Δf, is shown in Fig. 1(d) with no fit parameters. This family of curves, plotted for the same four values of Δf as used in the experiment (with the same color scheme), satisfactorily reproduces the measured angular width of the BER, as well as the weak dependence on data rate.

Supplementary Reference

[1] M. Schwartz, W. R. Bennett, and S. Stein, *Communication Systems and Techniques*. IEEE Press, 1996.
