# Supplementary material for: Frequency-division multiplexer and demultiplexer for terahertz wireless links
Source: Nat Commun. 2017 Sep 28;8:729. doi: 10.1038/s41467-017-00877-x (PMC5620079; doi:10.1038/s41467-017-00877-x)
Supplement: Supplementary file 2 — Description of Additional Supplementary Files [file 41467_2017_877_MOESM2_ESM.pdf]

Description of Additional Supplementary Files

File Name: Supplementary Movie 1
Description: The video shows real-time demultiplexing of two independent data channels, containing two different television broadcasts. As the receiver is moved between two different angular positions, the signal displayed on the monitor switches between the two demultiplexed channels. This demonstrates the long-term stability and error-free operation of the demux device.
